# The Rise of Wearable Devices during the COVID-19 Pandemic: A Systematic Review †

**DOI:** 10.3390/s21175787

**Published:** 2021-08-28

**Authors:** Asma Channa, Nirvana Popescu, Justyna Skibinska, Radim Burget

**Affiliations:** 1 Computer Science Department, University POLITEHNICA of Bucharest, RO-060042 Bucharest, Romania; 2 DIIES Department, University Mediterranea of Reggio Calabria, 89100 Reggio Calabria, Italy; 3 Department of Telecommunications, Brno University of Technology, 61600 Brno, Czech Republic; skibinska@feec.vutbr.cz (J.S.); burgetrm@feec.vutbr.cz (R.B.); 4 Unit of Electrical Engineering, Tampere University, 33720 Tampere, Finland

**Keywords:** COVID-19 pandemic, wearable devices, real-time monitoring, physiological monitoring, sensors

## Abstract

The COVID-19 pandemic has wreaked havoc globally and still persists even after a year of its initial outbreak. Several reasons can be considered: people are in close contact with each other, i.e., at a short range (1 m), and the healthcare system is not sufficiently developed or does not have enough facilities to manage and fight the pandemic, even in developed countries such as the USA and the U.K. and countries in Europe. There is a great need in healthcare for remote monitoring of COVID-19 symptoms. In the past year, a number of IoT-based devices and wearables have been introduced by researchers, providing good results in terms of high accuracy in diagnosing patients in the prodromal phase and in monitoring the symptoms of patients, i.e., respiratory rate, heart rate, temperature, etc. In this systematic review, we analyzed these wearables and their need in the healthcare system. The research was conducted using three databases: IEEE Xplore^®^, Web of Science^®^, and PubMed Central^®^, between December 2019 and June 2021. This article was based on the PRISMA guidelines. Initially, 1100 articles were identified while searching the scientific literature regarding this topic. After screening, ultimately, 70 articles were fully evaluated and included in this review. These articles were divided into two categories. The first one belongs to the on-body sensors (wearables), their types and positions, and the use of AI technology with ehealth wearables in different scenarios from screening to contact tracing. In the second category, we discuss the problems and solutions with respect to utilizing these wearables globally. This systematic review provides an extensive overview of wearable systems for the remote management and automated assessment of COVID-19, taking into account the reliability and acceptability of the implemented technologies.

## 1. Introduction

COVID-19 is a contagious respiratory illness caused by SARS-CoV-2. SARS-CoV-2 spreads from one individual to another through droplets emitted [1] when an infected person coughs, sneezes, or talks or the individual inhales infectious aerosols. It might likewise be spread by indirect transmission via fomites (contaminated surfaces) [2] to the hand upon contact and from hands to the mucous membranes on the face, as people touch their faces frequently. The most common signs and symptoms of COVID-19 are fever, cough, and trouble breathing. Fatigue, muscle pain, chills, headache, sore throat, runny nose, nausea or vomiting, diarrhea, and a loss of taste or smell may also occur [3]. The signs and symptoms may be mild or extreme and usually appear 2–14 d after exposure to SARS-CoV-2 [4]. Some people may not have any symptoms, but are still able to spread the virus. Most people with COVID-19 recuperate without needing special treatment [5]. However, other people are at higher risk of serious illness. Those at higher risk include older adults and people with serious clinical issues, such as heart, lung, or kidney disease, diabetes, cancer, or a weak immune system [6,7]. Serious illness may include life-threatening pneumonia or organ failure. Research is being performed to treat COVID-19 and to prevent infection with SARS-CoV-2.

The World Health Organization (WHO) declared the outbreak a public health emergency of international concern on 30 January 2020 and a pandemic on 11 March 2020, addressing a comprehensive multisectoral approach to prevent further spread. The word pandemic comes from two words, pan and demos. Pan means everyone, and demos means the people. A pandemic is an epidemic that happens on a scale that reaches past international boundaries and affects everybody on the planet. A disease or an illness cannot be a pandemic if it is not widespread; to become a pandemic, it must be very infectious. A pandemic such as COVID-19 might have started from animals with the virus, and then, the animals infected people, then people transferred the virus to the point where it has spread worldwide. However, from various research studies, it is clear that the WHO has no official “pandemic” category [8]. Globally, as of 5:14 p.m. CEST, 17 June 2021, there have been 176,693,988 confirmed cases of COVID-19, including 3,830,304 deaths, reported to the WHO [8].

Medical and nonmedical teams worldwide are actively looking for solutions to inhibit, mitigate, and slow the spread of SARS-COV-2 [9]. In the last two decades, we have seen wearable technology grow enormously, especially in the healthcare sector [10]. Wearable sensor technology in parallel with medical-grade wearable devices brings a unique opportunity to alter the one-time, fixed-assertion viewpoint of prediction to a real-time and objective approach for the prodromal-stage detection of a disease’s evolution. First of all, wearables are practical and passive, capable of continuously monitoring with little input from the users. Secondly, they can be implemented easily and work efficiently in inpatient or remote settings, hence providing a noninvasive assessment of patients. Lastly, wearables have the capability to present objective measurements of physiological parameters that may correlate with feasible wireless network systems and serve as a platform for real-time feedback to patients and doctors. Moreover, wearables are paving the way toward becoming more advantageous with respect to the pandemic. For example, Reference [11] developed a paper-based electrochemical biosensor for diagnosing COVID-19, and this device is capable of detecting targeted antibodies, with a sensitivity and specificity of 100% and 90%, respectively.

Wearables can provide a key early-warning system about the likelihood of COVID-19 infection, but their use can potentially go further in infection surveillance. There have been wearable devices introduced in the literature that not only make physiological measurements, but also focus on contact tracing applications for disease prevention. The potential of wearables in healthcare is enormous, but there are a number of challenges that need to be overcome, some of which are technical, some social, and others political, such as: few validation studies have been conducted, which creates obstacles for healthcare workers regarding the clinical utility of wearables; false-positive results create risks, so greater work and effort are needed for the correct interpretation of the data; patients are always concerned with the privacy and security of their data, as most data can be shared in the event of the breaching of the digital network’s security. Already, there are many wearables in the form of fitness trackers, smartwatches, and smart helmets available with built-in sensor technology to monitor COVID-19 symptoms. However, these gadgets only follow one or a few related symptoms. There is also a great need for existing technology to meet other demands such as cloud-based solutions for remote monitoring of patients.

Our paper’s contribution to this field is twofold:

- First, we present a comprehensive review on ehealth wearables for COVID-19, emphasizing their data interpretation models based on machine learning (ML) and deep learning (DL), the types of devices that have been used until now and that have arisen over time, and the parameters they can measure. Then, we also analyze the cloud/edge/fog environments used in wearable-based solutions, the different application areas of these wearables the context of the pandemic, and finally, the position and diversity of devices attached to the body to record important signals;
- Secondly, we address the problems and solutions with respect to using wearables in the healthcare system regarding the social, technical, and political aspects.

The structure of paper is as follows. The research methodology selected for this systematic review is depicted in Section 2. Section 3 elaborates state-of-the-art in ehealth wearables. Section 4 presents a detailed discussion of the utilization of already-existing wearable devices and the proposed devices’ adaptability on medical and clinical grounds. Finally, the summary of the review is drawn in Section 5.

## 2. Methodology

**Figure 1:**
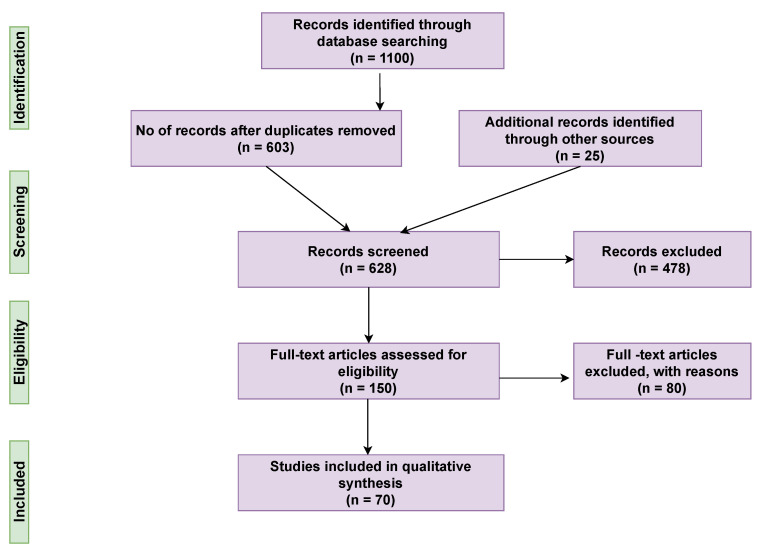
PRISMA-based flow diagram used for articles’ systematic selection.

This systematic literature review was carried out using the PRISMA guidelines [12]. Initially, we selected three databases for our present study, which were: IEEE Xplore Digital Library [13], PubMed [14], and Web of Science (WoS) [15]. The next step was to adopt appropriate keywords and their combination, and we chose “COVID-19” and “wearables”. In order to search all the related articles with these two main keywords, we proceeded with advanced research tools and adopted the Boolean expression AND and the asterisk (*) sign. Hence, the final keywords were: COVID-19 AND Wearable* for a publication period between 2019 and 2021, as the pandemic occurred during this timeline. After the complete search of the important databases based on this expression, we came up with 1100 articles; in parallel with these, we added 25 more records from other sources as shown in Figure 1. In the second stage, we initially removed 500 duplicates since the same articles were found in different databases. In the next stage, the articles were filtered out based on the title and abstract, and hence, 150 articles were screened out. In the fourth stage, the full-text articles were assessed for eligibility, out of which 80 articles were excluded, and the remaining 70 articles were included in the core and discussion of this systematic review on wearable devices and their rise during the COVID-19 pandemic.

## 3. Research Directions in Terms of Wearables for the COVID-19 Pandemic

This section presents the comprehensive review of the wearable devices and unobtrusive sensing technologies that are able to monitor the early symptoms of COVID-19 and common health conditions and the telehealth framework for the remote screening and diagnosis of disease and highlights unobtrusive sensing technologies that can be used in ubiquitous in-home and public-domain monitoring. The types of commercially available wearable sensors and devices that help in the diagnosis of early symptoms are addressed, and the proposed studies are discussed, which are still at the testing phase. For telehealth monitoring systems, the role of cloud, edge, and fog computing is discussed. These wearables are also capable of helping to prevent COVID-19 by maintaining social distancing and contact tracing. Other than the use of wearable in healthcare, wearables are now being adopted in smart learning tasks.

### 3.1. Wearable Sensors (Devices)

As technology is growing rapidly and becoming a part of our daily lives, people are relying more on technology with each passing day [16]. Currently, people wear a number of wearable devices, from fitness trackers to smart glasses, smart rings, smart shoes, smart contact lenses, etc. In the last decade, we have seen an unpredictable rise in smart wearables. With the ever-growing popularity and implementation of wearables in sensing physiological signs, many devices have been introduced into the healthcare system to provide more robust results [17]. Wearables provide ubiquitous, personalized services to the end users [18] and are equipped with a range of sensors. The availability and prevalence of these devices are what separate the present situation from that of the past. These devices have built-in features that allow them to have good and efficient use. The technologies measuring COVID-19 symptoms such as temperature, oxygen saturation level, or breathing rate already exist. Many companies and startups are endorsing these wearables and modifying them with cloud-based infrastructure to meet the need of the remote assessment of COVID-19 patients. Meanwhile, the problem with these solutions is that the gadgets they use can only measure or track one or two symptoms of COVID-19. In [19], the startup MyHomeDoc offered a remote monitoring system consisting of four embedded sensors, which connect to the user’s smartphone to provide vital signs instantly and remotely. Similarly, Reference [20] presented a wearable device that monitors heart rate (HR), stress level, sleep pattern, pulse oximetry, activity tracking, and other features in one device. Clinicians are able to assess patients remotely on a cloud platform. Reference [21] introduced a novel wireless pulse oximeter for oxygen saturation and respiratory rate (RR) system based on a cloud platform. Subject data are transferred from the wearable device using Bluetooth to an Android app, which are further sent to the hospital for testing. Similarly, in [22], the Vital Patch company offered a at-home monitoring system that monitors temperature, pulse rate, oxygen range, and respiratory rate (RR) for seven days.

The aforementioned proposed systems are hybrids of healthcare medical devices and commercially available gadgets. Most of these need to be set up by a hospital, for example the measuring devices, data centers, and display units, and involve maintenance and running fees, which obviously make these systems higher in price and less affordable to the public. Apart from the implementation of these systems, there is the need for medical device approval or other regulatory aspects such as approval from the U.S. Food and Drug Administration (FDA). Moreover, a system providing multimodal sensing of all the symptoms of COVID-19 and helping in its prevention has rarely been seen. The integration of wireless devices with sensors and transducers into wearable systems is becoming more common due to progress in microfabrication and nanofabrication technologies. Here, sensors with applications for detecting the symptoms of COVID-19 and their positions are reviewed.

#### 3.1.1. Types Of Sensors

1. Temperature sensors: For COVID-19, fever is the most common symptom, making temperature sensors a critical component of a wearable sensing system. According to the review article [23], in 90% or more of cases, fever is the main clinical representation of COVID-19. Hence, monitoring fever is immensely important for diagnosis. Apart from this, its continuous monitoring can give insights into the cause and nature of the disease, which would aid in better estimation of the care and treatment needs. For COVID-19 decision-making, temperature measurement is crucial, and temperature sensors play a vital role. Commercially available temperature-sensing devices comprising temperature sensors and approved by the FDA are elaborated in Table 1. TempTraq [24] is an interesting sensing device that detects infants’ temperature and sends the data to a smartphone app. TempTraq is a soft and comfortable patch that continuously monitors temperature for around 48 h. The sharing of the data with the mobile app is performed using Bluetooth. Another similar commercially available wearable device is the oura smart ring [25], which records body temperature, step count, and heart rate (HR). The ring has a good battery life, lasting up to seven days. The ring is water-resistant and weighs 7 g. The readings can be checked using the mobile app via Bluetooth. Another wearable device is Fever Scout by VIvaLNK in the form of a thermometer patch [26] that records fever wirelessly. Numerous low-power temperature sensors are available with different structures and calibration methods, as illustrated in Table 1. Most are built using MOSFET technology, having a BJT-less temperature-to-frequency/digital structure, taking advantage of the low-power design by using subthreshold MOSFET transistors and removing the need for external clocks and power-consuming ADCs. This also depends on the number of features the device offers, for example reusability and remote data sharing, whether it is power hungry, and the battery life.
2. Pulse oximeters: One of the significant processes of the human body is transporting oxygen by hemoglobin through the circulatory system. A lack of oxygen, i.e., SpO_2_, can cause brain damage, heart failure, or sudden death if it reduces to less than 95% [27]. To avoid this situation, pulse oximeter sensors play a very important role, as they obtain the photoplethysmogram (PPG) and determine the blood oxygen saturation level based on the light absorption characteristics of oxygenated and deoxygenated hemoglobin. Typical measuring sites are the finger, the toes, and the lobe of the ear. Most sensors, however, are located at the finger tip. References [28,29,30] introduced commercially available pulse oximeter devices, while [31] introduced a battery-free miniaturized fingernail wireless pulse oximeter, as explained in Table 1. Table 1 depicts the features of commercially available wearables, as well as the existing research methodologies adopted. The features that differentiate one device from other are long-term monitoring technology, battery life, the reusability of the device, as well as multimodal symptom detection. This points to the need to have a device that is reusable, has a long-lasting battery, measures multiple parameters, and is available to the general public.
3. Respiratory rate: Changes or anomalies in the respiratory rate of a patient also help determine the progression of an illness. Together with SpO_2_, HR, and body temperature, RR is one of the clinical features for evaluating the severity of a respiratory disease, e.g., a patient with severe respiratory distress has an RR greater than 30 breaths/min, which can develop into acute respiratory distress syndrome (ARDS) [32,33]. However, for COVID-19, RR can serve as a vital prognostic factor. Wearable strain-gauge sensors, triboelectric sensors, and accelerometers have been extensively studied to detect respiratory movement in the thorax or abdomen caused by respiratory volumetric changes [34]. The wearable technologies include thermal, humidity, acoustic, pressure, resistive, inductive, acceleration, electromyography, and impedance sensors. A wearable device developed with these sensors can be attached to chest belts [35,36] or mounted to the skin [37]. Some of the wearable RR-monitoring products are RespiraSense [38], Spire [39], and epidermal thermal sensors as in [37]
4. Cough and lung sound monitoring: Dry cough is one of the symptoms of COVID-19. People infected with COVID-19 may spread the disease when they cough. Therefore, the monitoring of dry cough not only helps in the diagnosis and progression of the illness, but also helps in its prevention. Cough signals are typically acquired with an audio or mechanical sensor that can detect the coughing sound or the vibration caused by the cough, respectively. Such sensors include a microphone that can be wearable or a piezoelectric transducer and a highly sensitive accelerometer that can be mounted at the throat or the thoracic area [40,41,42]. With audio signal processing and pattern recognition approaches such as ML classification algorithms, cough can be identified automatically [41]. Auscultation of the lungs is an important part of respiratory examinations. In [43,44], the authors proposed a wearable stethoscope patch that combines sensing modalities such as a MEMS stethoscope, ambient noise sensing, ECG, impedance pneumography, and nine-axis actigraphy. The system is able to perform auscultation continuously without requiring the distribution of sensors over different places of the body, to detect wheezing or other adventitious respiratory sounds.
5. Electrocardiogram for monitoring COVID-19 patients: ECG is a diagnostic tool used to assess the activity of the heart and provide the risk assessment of COVID-19 treatment. Wearable-based tele-ECG monitoring instead of the traditional ECG monitoring systems used by medical practitioners can potentially reduce cross-infections by reducing staff-to-patient contact. Adhesive ECG patches are one of the most common wearable ECG monitoring approaches. The ECG patch device typically consists of a sensor system, a microelectronic circuit with a recorder and memory storage, and an internal embedded battery. These patches are small in size, wireless, with miniaturized electronics, easy to wear, and comfortable to use and can record ECG for many days. For example, the MCOT patch [45] (BioTelemetry, Malvern, PA, USA) is used to monitor the ECG of patients treated with hydroxychloroquine and azithromycin. Other ECG patch products with a similar function have been used in clinical studies including the Savvy monitor (Ljubljana, Slovenia) [46], the SEEQ MCT patch (Medtronic, Inc., Dublin, Ireland) [47] designed, developed and launched by Corventis, Inc. of San Jose, CA, USA, and the VitalPatch wearable sensor (VitalConnect, San Jose, CA, USA) [48].
6. Blood pressure monitoring: Blood pressure (BP) is one of the most important vital signs that reveals cardiovascular and cerebrovascular functions. High BP, called hypertension, is the main risk factor for cardiovascular morbidity and mortality. The vulnerable population, i.e., those with underlying conditions, has a higher risk of severe complications from COVID-19 [49,50]. BP is usually measured by cuff-based sphygmomanometers by medical staff, which significantly increases their work load and the possibility of them becoming infected. According to [51], COVID-19-positive patients experience a sudden fall in BP, presumably due to the “cytokine storm”, which is the disastrous overreaction of the immune system. Hence, continuous and remote monitoring of BP in real time may help to prevent sudden events and reduce the possibility of cross-contamination. Some of the unobtrusive BP-monitoring wearables proposed are BP watches [52], BP eyeglasses [53], flexible BP patches [54], BP shirts [55], and wearable skin-like BP patches [56]. Although the research on continuous and unobtrusive monitoring is much more advanced, there are still some obstacles that need to be overcome, especially the accuracy when tracking responses to medications. Because of the dynamic nature of BP and its variability in different individuals, it is challenging to obtain accurate BP estimations for a long time without calibration.

#### 3.1.2. Position of Sensors

**Figure 2:**
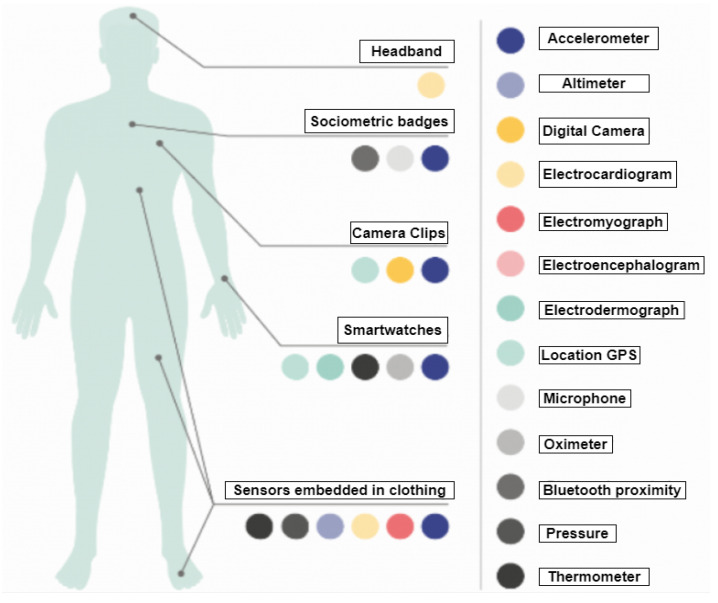
Wearables based on on-body location [57].

In our critical analysis of wearable sensors, one important factor we want to highlight is the importance of the on-body position and the number of wearables. Aggregate data taken from wearables can also contribute to the research by detecting general patterns and trends within a population, which can contribute to improved public health responses. Cumulative data can also be used to identify geographical COVID-19 hotspots. As mentioned before, for a symptom such as decreased SpO_2_, this is mostly acquired from the finger tip, ear lobe, or toes, but if focused more in a systematic way, we could find a standardized solution such that we can solve the many issues of the design tradeoffs, power consumption, computational errors, cost issues, and many more. From Figure 2, it is clearly seen that sensors such as accelerometers, EMG, ECG, altimeters, pressure sensors, and thermometers can be embedded in clothes and can record multiple physiological parameters such as motor activities, small electrical signals generated by muscles, electrical impulses through the heart muscle, a person’s location/distance, the vertical ground reaction force while walking, and fever. Similarly, a smartwatch can have diverse sensors and measure multiple parameters such as EEG signals, the location of a person, step counts, body temperature, SpO_2_, EMG, and ECG.

### 3.2. Use of Artificial Intelligence for the Diagnosis and Prevention of COVID-19

Strategies employed using artificial intelligence (AI) and deep learning (DL) approaches can speed up the screening of the spread of the virus, aid in distinguishing mild to severe infections, and be used in supervising the disease continuously. These become even more powerful when the correct technique is applied on the right data from the right devices. When the pandemic started, researchers’ main motive was to forecast its spread, as seen with the Johns Hopkins COVID-19 dashboard [58]. Then, research focused on screening and diagnosis, as in [59]. Here, the authors used smartwatches to collect data from patients and used a heuristic model for its detection. In the context of a pandemic, AI is applied in two main areas, namely medical research and the social context [60]. However, now, the role of AI is multifold, and it is capable of identifying who has the most risk, diagnosing patients, developing drugs faster, finding existing drugs that can help lower the spread of the disease, understanding viruses better, mapping where viruses come from, and predicting the next pandemic.

Machine learning (ML) has performed phenomenally in predicting risk factors. Similarly, for COVID-19, there are numerous risk problems where ML prediction and forecasting models can be very useful, and some of the problems are as follows:

1. Infection risk: Is a particular group of people or an individual at a high risk of getting COVID-19? This risk can be attenuated when the following statistics are provided in the right manner, i.e., age, current health condition, general hygiene habits, social activities, number of outdoor meetings, frequency of interactions, location, and climate;
2. Severity risk: It is always good to be on the safe side and stay away from complications that would result in the need for intensive care. Hence, healthcare practitioners need a system that predicts beforehand severe COVID-19 symptoms that would require hospitalization. Many individuals experience mild symptoms and some acute respiratory distress syndrome, which is certainly deadly, so it is better to begin treatment earlier if the symptoms are becoming worse. This can be solved by ML models, but some groundwork is needed, i.e., more data;
3. Outcome risk: With the surge in cases and the increase of the severity of the symptoms in an individual, it is necessary to know the treatment’s outcome, which literally means knowing whether a patient would survive or not. This way, doctors will be confident and able to effectively treat patients. Since treatment methods for COVID-19 are still evolving, there is still some time before AI plays a role in this field, but similar work has been performed in outcome prediction in patients with epilepsy [61];

- Using wearable technology along with AI: At the start of the pandemic, the Apple and Fitbit [62,63] smartwatches made headlines regarding the following and tracking of COVID-19 symptoms; at that time, the research was still young, but now, researchers are using better computational algorithms, and have proven that the use of wearables along with AI gives promising results. If we take the process of diagnosing a viral infection, there is a high probability that the person who takes the sample from the patient may also become infected. The testing results take a few hours, and the person can transmit the virus to a group of people during this time. To avoid these problems, medical staff remotely monitor the patient’s BP, ECG, pulse rate, HR, and fever using wearable devices with AI technology. We summarize the work performed in the literature using AI technology during the COVID-19 pandemic in Table 2.

Machine learning is an important tool in fighting the current pandemic. If we take this opportunity to collect data, pool our knowledge, and combine our skills, we can save many lives—both now and in the future. However, this requires great support from the community, IT professionals, wearable sensor companies, healthcare institutes, policy-makers, and researchers.

#### Role of Cloud, Edge, and Fog Computing along with Wearables to Mitigate COVID-19

IoT technology connects devices with each other, and with an Internet connection, it provides a better and meaningful association of everyday things and people [70]; this is also why it is called the Internet of Everything (IoE). The failures of the healthcare systems even in well-developed nations during the pandemic have been due resources not being well developed and managed. Patients needed to travel from home to the hospital for diagnosis and testing, and they made consequent visits to their doctors for assessments and check-ups. The concept of wearable technology has existed during this time, but the resource management techniques of the cloud, edge, and fog computing environments have been lacking. First of all, we elaborate on what cloud, edge, and fog computing mean:

- Cloud computing is undoubtedly one of the key research subjects for the past several years. It allows users to move their data and applications to the remote “cloud” and then access them in a simple and pervasive way [71]. A computing cloud is a set of network-enabled services, providing scalable, quality of service (QoS)-guaranteed, normally personalized, inexpensive computing infrastructures on demand;
- Edge computing is undoubtedly the main computing paradigm of the last decade. According to [72], “Edge computing refers to the enabling technologies allowing computation to be performed at the edge of the network, on downstream data on behalf of cloud services and upstream data on behalf of IoT services”. Basically, the idea is to extend cloud computing to the network edge with the aim of the computation being performed in the proximity of the data sources, i.e., IoT devices. This layer can be implemented in different ways. However, all the different implementations have been designed with the edge paradigm in mind; therefore, many similarities are present. The edge computing principles can be put in practice in several ways, in terms of the types of devices, the communication protocols, and the services;
- Fog computing provides distributed computing, storage, control, and networking capabilities closer to the user [73]. It is not just an another implementation of edge computing, but rather the highest evolution of the edge computing principles. Indeed, fog computing is not limited to only the edge of the network, but it incorporates the edge computing concept, providing a structured intermediate layer that fully bridges the gap between the IoT and cloud computing. In fact, fog nodes can be located anywhere between end devices and the cloud [74]; thus, they are not always directly connected to end devices. Moreover, fog computing does not only focus on the “things” side, but also provides its services to the cloud. In this vision, fog computing is not only an extension of the cloud to the edge of the network, nor a replacement of the cloud itself, but rather a new entity working between the cloud and the IoT to fully support and improve their interaction, integrating the IoT and edge and cloud computing.

**Figure 3:**
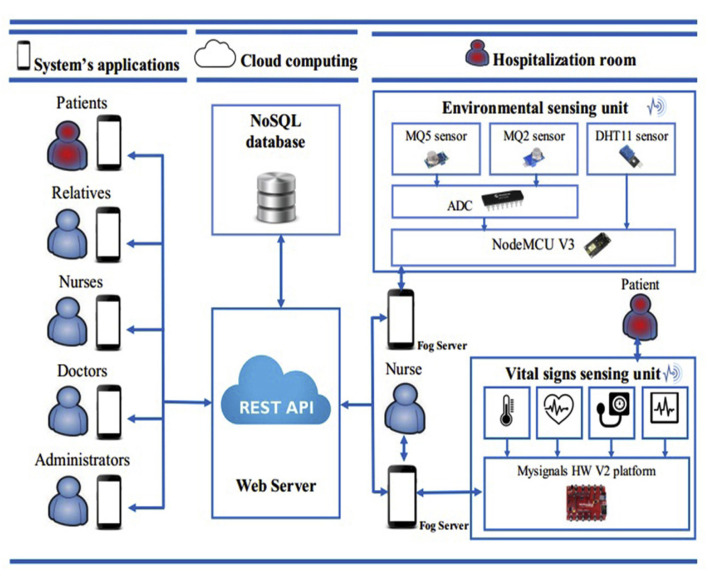
The home hospitalization platform proposed by [75] based on fog computing, which is a highly suitable and efficient system in the context of the COVID-19 pandemic.

In this study, we highlight the proposed research work based on the cloud, edge, and fog techniques along with the IoT-based wearable technologies that can make our healthcare systems more robust against any future pandemic. For instance, Reference [75] introduced a home hospitalization system based on fog computing, in which the patient (probably COVID-19-positive) has an on-body wearable device from MySignal that recorded the vital signs and connected wirelessly to the healthcare staff’s smartphones. Therefore, the doctor can continuously check the physiological parameters of the patient. Apart from this, there are environment sensors that are placed in the room of the patient, as shown in Figure 3. The signals from these sensors travel wirelessly to a smartphone via the fog and are also stored on a webserver via a cloud platform; the information in the cloud can be accessed by the patient, nurse, doctor, and relatives. Another work based on cloud and fog computing was performed by [76]. The architecture comprises a sensor network, a smart gateway, cloud processing, and behavior detection. Another novel approach was introduced in [77].

These proposed frameworks from the literature are robust at providing quality services and bringing significant improvement to the health sector by enhancing the recovery of patients, especially elderly people. Systems as the one presented in [75] are highly acceptable to patients, doctors, family members, and friends of the patient. In particular, in the times of COVID-19, IoT-based wearable technology has been continuously integrated with the cloud, edge, and fog paradigm. However, to strengthen these solutions, people need to be aware and educated about the use of IoT-based devices and mobile applications. Moreover, the implementation of the proposed systems meets some challenges that must be resolved in a timely manner, in lieu of expensive sensing units, which should rather be cost-effective so that low-income patients can acquire such systems. In addition, keeping in mind the corona virus situation, new features need to be added to these systems.

### 3.3. Applications

**Figure 4:**
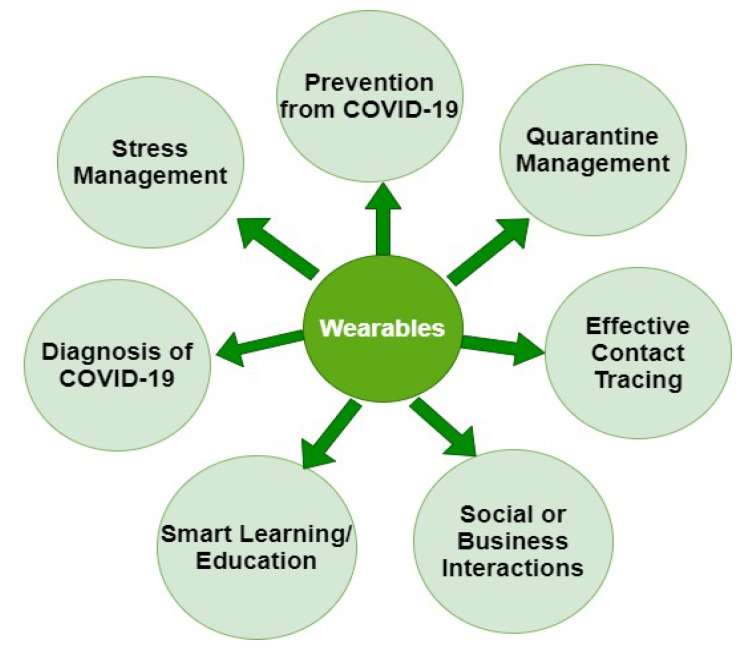
Potential applications of wearables in the COVID-19 pandemic.

Numerous consumer technologies have been developed for health and well-being during the COVID-19 pandemic, relating to different problems. Some of the main application areas are depicted in Figure 4. At the beginning of the COVID-19 pandemic, wearables were mostly used to track the symptoms of patients: fever, high heartrate, cough, and oxygen saturation level; but now, the applications of wearables are wide ranging. For example, Reference [78] proposed a smart mask assembled with wearable sensors and some actuators that detect airborne pathogens and also take necessary measures to mitigate them. Reference [77] introduced the use of multiaccess edge computing (MEC) over the edge computing paradigm to provide a basis for contactless treatment in order to prevent COVID-19. This novel system provides various services, scalable access to all IoT medical devices with improved link capacity, and the advantages of the storage and processing resources of the edge paradigm. Reference [79] presented a solution to estimate if the outdoor environment is empty or not. If not, then it determines the density of people using a cost-effective and nonintrusive device. To prevent the COVID-19 pandemic from worsening, it is very important that COVID-19 electronic medical records (CEMRs) among hospitals all over the world be shared, while considering patient privacy. To deal with this aspect, a blockchain-based medical research support platform was introduced by [80]. It keeps track of records and updates the system automatically while sharing the information in the most secure way. Hence, the joint efforts of all countries around the globe to control COVID-19 can provide efficient and privacy-preserving data sharing [80].

Although healthcare is still the fastest-growing category, new wearables have the potential to grow in the areas of contact tracing, remote treatment of patients, leisure activities during quarantine, and continuous care. However, new challenges and further questions are still presenting themselves. Researchers must modify these systems for practical implementations. As observed from the literature, less work has been performed in prediagnosis. Reference [81] made use of a mobile app and wearable sensors for the early diagnosis of COVID-19 in students. The data were collected for one year, and the system has still not been validated. The same situation can be mentioned in the case of the post-COVID-19-infectioneffects on patients: this area needs more attention and work from researchers.

#### 3.3.1. Symptom Screening and Tracking

**Figure 5:**
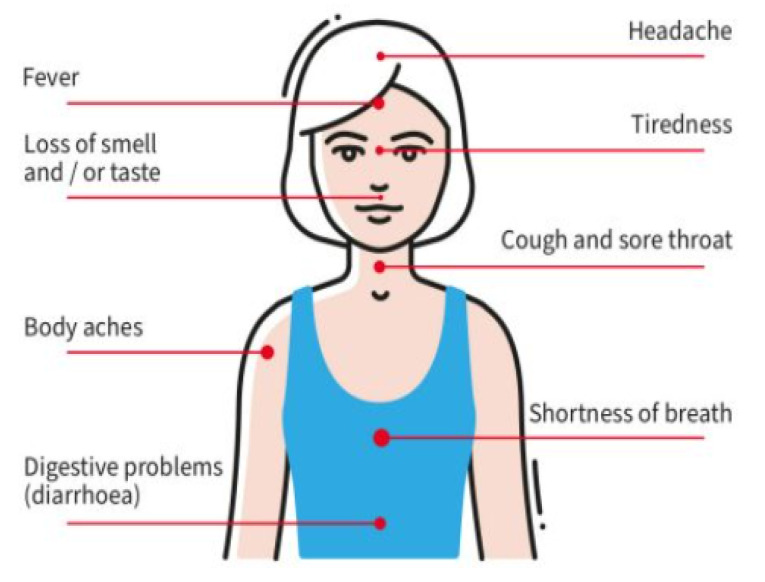
Symptoms of COVID-19.

The most common symptoms of COVID-19 are: dry cough, fever, muscle ache, fatigue, and shortness of breath, as depicted in Figure 5. Along with these, other less-observed symptoms are diarrhea, headache, and hemoptysis. The subject who possesses all these conditions is a person infected with the COVID-19 virus. As time passes, the virus eventually affects the lungs’ functionality with the impact increasing up to 14 days. Among the symptoms, research has found that body temperature and dry cough are the vital diagnosis parameters of COVID-19. In Table 3, we summarize the recent studies conducted in screening and tracking the symptoms of COVID-19, as well as the technologies that can be adopted to prevent people from becoming infected by this deadly virus.

#### 3.3.2. Use of Wearable Devices in Digital Contact Tracing and Social Distancing

We all are quite aware now that the COVID-19 virus turned into a pandemic when it spread globally. At this moment, people know that the probability of infection is reduced if they maintain their distance from others. If someone is notified of being COVID positive, then he/she has to go into quarantine, as well as anyone who might have been infected at that time. Contact tracing helps to find physical interactions between humans at distances of 1.5–2 m and also for a specific amount of time, i.e., two weeks. First, contact tracing was performed traditionally through interviews, which consumed much time and was full of errors. For example, it was very hard to recall everyone and ask the names of all persons with whom one came close to in the last week. Subsequently, tracking apps, mobile phones, wearables, and some powerful computational methods have joined hands to solve this issue, as well as providing solutions for maintaining social distance. Some of the recent research related to contact tracing and social distancing is discussed in Table 4.

Undoubtedly, digital contact tracing provides better results than traditional approaches. From the start of the pandemic, if digital contact tracing had been well developed and public health measures had been correctly followed, the morbidity and mortality rate would have been much lower than now. Until now, very few empirical studies evaluating the effectiveness of digital solutions for contact tracing have been performed. For example, References [96,97] presented the Si-CMOS optoelectronic micro-nano system technology, similar to the microwave photonics and RFs proposed by [101], for social distancing and contact tracing scenarios, which may enhance the technical aspect of the system. This area needs more attention for a proper implementation. While developing wearable devices for contact tracing applications, the researchers must follow data privacy regulations [102]. The accuracy of these tools for contact tracing and maintaining social distancing will subsequently reduce the burden [103,104] on governments and healthcare systems.

#### 3.3.3. Stress Management Using Wearables

Since the pandemic started, the mental health of many people has been affected, and this challenging situation has led to an upsurge in reports of pathological stress, depression, anxiety, and insomnia [105]. This public health calamity has changed people’s lives and affected their lifestyle, both at home and at work. Undoubtedly, the pandemic has put an additional pressure on healthcare practitioners around the world, as they are in direct contact with patients. In this area of mental heath management, stress level detection wearable technology has not been left behind. In [106], the authors introduced a stress detection system using wearable devices and a DL-based algorithm. In this study, 212 medical staff participated, and the data were collected for 10 weeks continuously using Fitbit smartwatches, smartphones, smart bras (OMSignal), audio recorders (TAR), and location data (Owl-in-one). Then, the data were interpreted using a long short-term memory (LSTM) deep neural network for stress detection, which produced good scores. However, the results may vary for employees working at night, and more physiological features can be included in the future.

In [107], the authors analyzed the stress level of a small group of Canadians who wore activity trackers and tracked their stress level during the initial month of the COVID-19 pandemic. The findings of the study provided good results. Similar to many past studies, it was concluded that sleep detection from smart wearables produces better accuracy than self-reported questionnaires. In [108], the researchers used pulse oximeters on clinical staff in the area of Wuhan to check the severity of their insomnia and mental health status. As social life and leisure activities have been badly effected during the pandemic, we have seen that students have more depression and stress. In this context, there are some consumer-grade wearables that can measure students’ anxiety and depression, as described in [109,110]. In this systematic review, we wanted to highlight that this deadly virus has affected the mental and emotional health of people very negatively. Wearable devices that are already commercially available such as fitness trackers and smart bracelets or the wearable devices that are going to be implemented in the near future can be utilized to intervene in patients’ psychological health. This will reduce the costs, if the design is developed especially for stress detection. The possibility of advancing and modifying wearable devices must be investigated.

#### 3.3.4. Smart Learning

Since COVID-19 started, educational institutes have been closed all across the world; the learning system has been drastically affected, and now, the status of education is changing dramatically. As a result, there is a rise in e-learning, whereby teaching is undertaken using digital platforms. The IoT has opened wide possibilities in the area of smart learning [111]. The use of smart wearables can make the education system during the pandemic more efficient and smarter. Although classrooms are organized with smart devices such as smart boards, it is still troublesome for teachers to check each student separately and find out where he/she needs more attention, as every person has a different learning pattern. Hence, assisting every student requires much time and effort, especially when the learning strategy has changed to online learning. Now, the research approach is to enhance the smart learning paradigm and teaching method by utilizing the IoT and the available wearable devices and sensors in combination with machine learning (ML) and artificial intelligence (AI).

In this context, Reference [112] conducted an experiment using an IoT device and an AI algorithm to identify students’ behavior in class and to check if they were able to understand the lecture or not or if they needed more attention from the teacher. Similarly, mobile-based apps and RFID- and NFC-enabled devices can help with a smart attendance system for institutes or tracking the location of teachers or the administration in case of an emergency. Additionally, the use of eye-worn devices, i.e., smart glasses [113], may help students document lectures, capture videos, learnin real time, prepare on-site reports, and have a real-world-like experience using augmented and virtual reality. In the pursuit of improving the learning system, in large classrooms with a huge number of students at the back of the classroom, most students cannot clearly see the board in front and hesitate to interact with the teacher, as this involves using a loud voice. Hence, smart screens [114] are built into the desks. This kind of system has proven to be very promising, and its implementation has been encouraged. In [115], a pen was developed that tracks how much time a student takes while solving a question on an exam. To develop a strong smart learning management system during the COVID-19 pandemic, it is highly important that higher education institutes and universities share their experiences and collaborate. Moreover, this implementation requires trained and skilled professionals in IoT-specialized subjects. Teachers and students need training on how to use online platforms and how to manage smart systems.

## 4. Potential Barriers to Wearables’ Usage and Their Solutions

With respect to our discussion, wearables have shown their potential in healthcare; however, there are various challenges that must be overcome. Most wearable technologies are still in their prototype stages. Issues such as user acceptance, security, ethics, and big data concerns with respect to wearable technology still need to be addressed to enhance the usability and functions of these devices for practical use. For better understanding, these barriers are classified below.

### 4.1. Potential Barriers

- Technical issues: Wearables comprise a relatively new technology. Therefore, the utility of wearables at the clinical level is still limited. Healthcare beneficiaries are withholding wearables’ implementation at the clinical level as there is a strong need for more validation studies. This problem can be resolved by the government’s and individuals’ commitment to clinical trials. Based on the feedthrough mechanisms in the clinical atmosphere, there is a possibility to gather huge datasets from various sources. False information is also possible, but real monitoring and processing systems can lessen such problems. This can also decrease the time that the patient needs with the medical practitioner and will help generate a highly integrated real-time healthcare system. There is a high risk of security breaching, which is the most common issue in security systems. This issue can be solved by addressing the points such as where the data from a given device are deposited, to whom the access is provided, and the duration the data are available. Data collection and storage are usually determined by the user. Therefore, the accountability for their usage is user-defined. Apart from this, the wearable system also should not affect the daily behavior of the patient, nor seek to directly replace healthcare professionals. The wearable devices should be compact and easy to use and wear. It has become apparent that despite the importance of user preferences, there is a lack of high-quality studies in this area. These issues become increasingly important if they seek to obtain measurements over longer time periods, for example in monitoring a patient during quarantine;
- Social interruption: Internet access and device penetration are not the same world-over, though the data accumulated from a demonstrative cohort can have a positive influence on the broader public. Provided the comparatively lesser price of a few devices, there should be a governmental allocation to front-line workforces and susceptible groups. Wearable devices require a higher level of digital knowledge, though automatic functions can alert the users. Wearables can be especially efficient in elderly care; however, this group is less skilled with technology. There is a possibility that the alerts might make people nervous, but their use is elective and does not disclose diagnosis. For various people, comprehending one’s personal health and infection possibility would be advantageous, and the wider social effects might be positive;
- Regulatory aspects: There are various barriers that stop the wearables industry from reaching an advance level of innovation. One of them is that each device requires intricate and lengthy procedures before approval. For instance, during the COVID-19 pandemic, the U.S. FDA distributed a new plan that permits manufacturers having FDA-identified devices to increase their utilization so that healthcare beneficiaries can apply them to monitor patients, remotely. Recently, Apple watch’s ECG function has gained permission from the U.S. FDA and nineteen European controllers. Within the European Union, the delivery of new medical devices has been delayed as a result of the COVID-19 crisis.

### 4.2. Solutions

Considering the technical and design issues, user preferences need to be considered to design these wearable devices, which will gain acceptance both in the clinical and home setting. A body-worn sensor system should be compact, embedded, and simple to operate and maintain. Researchers should be encouraged to focus on the implications of user preferences when designing wearable sensor systems. It has been observed that not many elderly people are currently using wearable devices because, generally, there is a lack of technological awareness among older generations. First, there is a need to test wearable devices to determine if they meet the needs of elderly people, and then, technological awareness among the elderly population must be promoted. For data security and patient confidentiality, security must be evaluated in these devices before implementation. For the data interoperability challenges, the fifth-generation of wireless networking technology (5G) will enable us to connect many times more hospital devices to the network at once and to gain remote access at home. Given the great adoption of wearable technologies in all aspects of human life, the legal, regulatory, and policy issues concerning wearable technologies will have to be addressed in a distinct manner.

## 5. Conclusions

From the COVID-19 pandemic, we have clearly realized that if health systems had accelerated the adoption of technology available over the past few years, the magnitude of the current pandemic would likely have been much less severe. At present, we find there are already many wearables commercially available on the market; a number of solutions are at testing phase, and numerous wearables have been proposed by researchers. With digital solutions moving towards low power consumption and small-form-factor devices, multisensors may cover diverse physiological and contact tracing parameters, creating digital databases and providing access to medical practitioners, using cloud or edge services to analyze the effect of treatment or assessing the patients. This study presented how wearables have grown during the COVID-19 pandemic and still growing wherever there are demands that need to be met. The wearables must have multifunctional capabilities and be easily configurable for the desired end use application. Wearables with a single functionality (e.g., measuring only the heart rate) are useful, but in practical applications, more than one parameter is typically monitored; having multiple wearables, one for each function or data stream, would make the individual look the same as a cyborg and deter their use even if the multiple data streams could be effectively managed. The challenges related to the design trade-offs, improved sensors, power, size, computation algorithms, and security need to be resolved as soon as possible for the clinical utility of these wearables.

It is clear that in developing such solutions to prevent COVID-19, contributions from a wide range of fields such as biology, electronics, computer science, etc., are required. From the pandemics of the last 100 years, the viruses seem to be of different types and novel, but the patterns and symptoms are similar. This means that in the future, there will be the possibility that the world will face another virus. Contagious respiratory illnesses still remain a threat to our well-being in the modern world, and we should be ready to address this threat.

## Figures and Tables

**Table 1:** Wearables for monitoring body temperature and blood oxygen level.

Name	Category	Measurement	Wearability	FDA-Cleared	Reusable	Price
TempTraq [24]	Body temperature	Fever	Patch	Yes	No	USD 45
Oura ring [25]	Body temperature	Fever, steps, HR	Ring	Yes	Yes	USD 299
Fever Scout [26]	Body temperature	Fever	Patch	Yes	Yes	USD 20
CONTEC [30]	Pulse Oximetry	SpO_2_, PR, HR	Wristband with finger clip	Yes	Yes	USD 112
Battery-free pulse oximetry [31]	Pulse Oximetry	HR and HR variability	Finger nail	No	Yes	NA
MightySat [28]	Blood oxygen level	SpO_2_ level only	Finger clip	Yes	No	USD 299
PO3M [29]	Pulse oximetry	HR	Finger Clip	Yes	Yes	NA

**Table 2:** Summary of some research studies that used AI technology in the diagnosis and prevention of COVID-19.

Data	Modality	Results	Reference
Chest images	X-ray	In this study using deep-nets, COVID-19 was diagnosed with an accuracy of 91.67% and an accuracy of 100% in finding the survival ratio.	[64]
Clinical, laboratory, and radiological	Medical records (all history)	70% to 80% accuracy was achieved in predicting acute respiratory distress syndrome (ARDS) severity	[65]
Clinical	Historical medical claims data	Helps curtail the worst effects	[66]
RR, cough, temperature, accelerometry	Wearable patch	Detects cough related activity, seismocardiogram and RR observation using AI	[67]
Oscillating magnetic field	Wearable belt	Tracing: proximity estimation up to 0.1m	[68]
Step counts, sleep times, RHR	Stanfordwatch	80% early detection rate	[69]

**Table 3:** Summary of recent research studies performed on the screening, tracking, and prevention of COVID-19.

Wearable Device	Sensors	Multimodal Sensing	Remote Monitoring	Features	Study
Smart Telehealth-IoT system	Uses a body area sensor network (BASN) incorporated with a mesh of wireless sensors	✓	✓	Monitoring vitals: PPG, ECG, EMG, ACG, and AMG, unusual patterns while breathing and allowing the physician to remotely assess them	[82]
H-watch	Multiple sensors	✓	✓	Symptom monitoring and contact tracing	[83]
Headset	NTC thermistor, microphone, and PPG sensor	✓	✗	Respiration rate (RR), PPG, rapid or shortened breathing, and cough are detected	[84]
Smartwatch and a wearable accessory	Smartwatch sensors	✗	✗	Prevention from undesired face touching	[85]
Headset and mask	Thermistor, microphone, and PPG sensor	✓	✓	SpO_2_, RR, HR, temperature, and ECG	[86]
Wearable device	Pulse oximeter, HR sensor, temperature sensor, and vibration sensor	✓	✓	SpO_2_, HR, temperature, and hand movements to determine severity	[87]
Wearable mask	Optical fiber Bragg grating sensor	✓	✓	Respiratory rate monitoring	[88]
Smartwatch with an IMU module and a vibration motor	IMU sensors	✗	✗	Preventing touching the face	[89]
M5stickC device	Ambient sensor, infrared, and contact thermometer	✓	✓	Temperature monitoring and human activity recognition during quarantine	[90]
Oura ring	Infrared LEDs, accelerometer, gyroscope, and three temperature sensors	✓	✓	Diagnosis and prevention of COVID-19	[91]
Accelerometry-based device to prompt nonsupine positioning	Accelerometer sensors	✗	✓	Managing respiratory problems of COVID-19-positive patients	[92]
Oura smart ring	Skin temperature sensor	✗	✓	Onset of COVID-19 symptoms, i.e., fever	[93]
Smartwatch	Smartwatch sensors	✓	✓	Presymptomatic detection of COVID-19	[94]
Wearable in-ear (hearable)	Two PPG sensors	✓	✗	SpO_2_ measurement	[95]
Multimodal patch stethoscope	Single-lead ECG and impedance pneumography, 9-axis magnetic, angular rate, and gravity (MARG) sensors, digital stethoscope, and ambient sound recording	✓	✗	Estimation of ECG, PEP, LVET, and respiration	[44]

**Table 4:** Summary of recent research studies performed on contact tracing and social distancing in the context of COVID-19.

Wearable Device	Sensors	Multimodal Sensing	Wireless Connectivity	Features	Study
Fitbit, Garmin, Apple	Inertial and position-tracking sensors	✓	WiFi and Bluetooth	Social distancing	[96]
Wearable (no specific position)	Multiple sensors	✗	Bluetooth	Social distancing and contact tracing	[97]
Belt	Microcontroller with an ultrasonic sensor	✗	Bluetooth	Social distancing detection system	[98]
Smartphone	Inertial sensors, HR sensor	✓	Bluetooth	Contact tracing	[99]
Smartwatch and smartphone	Inertial sensors, vital sign monitoring sensors	✓	WiFi	Contact tracing	[100]

## Data Availability

Not applicable.

## Abbreviations

The following abbreviations are used in this manuscript:

IoT	Internet of Things
IoE	Internet of Everything
ARDS	Acute respiratory distress syndrome
AI	Artificial intelligence
ML	Machine learning
DL	Deep learning
PPG	Photoplethysmography
ECG	Electrocardiogram
EMG	Electromyogram
HR	Hear rate
RR	Respiratory rate
HRV	Heart rate variability
WHO	World health organization
5G	Fifth generation
FDA	Food and Drug Administration
LSTM	long short-term memory
